# Preoperative neutrophil-to-lymphocyte ratio and tumor-related factors to predict microvascular invasion in patients with hepatocellular carcinoma

**DOI:** 10.18632/oncotarget.19178

**Published:** 2017-07-12

**Authors:** Yanlong Yu, Jiuling Song, Ran Zhang, Zhonghua Liu, Qiang Li, Ying Shi, Ying Chen, Jinming Chen

**Affiliations:** ^1^ Department of General Surgery, Chifeng Municipal Hospital, Inner Mongolia Medical University institute of clinical, Chifeng 024000, Inner Mongolia Autonomous Region, China

**Keywords:** hepatocellular carcinoma, microvascular invasion, neutrophil-to-lymphocyte ratio, platelet-to-lymphocyte ratio, prognosis

## Abstract

Small hepatocellular carcinoma (HCC) is less invasive and has a better prognosis, but it still has a high recurrence rate. Microvascular invasion (MVI), as a poor prognostic indicator, is of great importance for treating of patients with HCC. The objective of the present study was to evaluate the predictive value of preoperative neutrophil-to-lymphocyte ratio and possible clinical parameters to MVI in patients with HCC. A total of 157 operable patients with HCC having a tumor diameter of less than or equal to 5 cm were enrolled in this study. The utility of neutrophil-to-lymphocyte ratio, platelet-to-lymphocyte ratio, and other clinical parameters was evaluated using receiver operating characteristic curves. MVI was identified as an independent influencing factor for disease-free survival in patients with HCC who underwent curative resection, using the multivariate Cox proportional hazards regression model. The independent parameters associated with MVI were determined using logistic analysis. Multivariate analyses indicated that the neutrophil-to-lymphocyte ratio [hazard ratio, 1.705; 95% confidence interval, 0.467–6.232; *P* = 0.022)], platelet-to-lymphocyte ratio (hazard ratio, 1.048; 95% confidence interval, 1.006–1.092; *P* = 0.025), and a-fetoprotein (hazard ratio, 1.012; 95% confidence interval, 1.003–1.021; *P* = 0.007) were significantly associated with MVI independently. Therefore, this study concluded that the preoperative neutrophil-to-lymphocyte ratio and a-fetoprotein might serve as useful biomarkers for predicting MVI in patients with HCC.

## INTRODUCTION

Hepatocellular carcinoma (HCC) is one of the most aggressive malignancies and a leading cause of cancer-related mortality [1]. This may be attributed to the rising trend of chronic hepatitis B virus (HBV) and hepatitis C virus (HCV) infections and incidence of nonalcoholic fatty liver disease [2]. HCC is the most common histological type of liver cancer. After a curative hepatic resection, the overall 5-year survival for HCC remains dismal despite improvements in screening and surveillance efforts for HCC [3].

The standard treatment for small HCC is still unable to reach a consensus compared with simple surgery or simple transcatheter arterial chemoembolization (TACE), radiofrequency ablation (RFA), and surgery-based comprehensive treatment, which have remarkable advantages and significantly improved survival time and local recurrence rate, especially for patients with microvascular invasion (MVI). For example, laparotomy after partial hepatectomy, supplemented with intraoperative RFA, is a radical treatment for patients with liver cancer having severe cirrhosis. This also avoids excessive removal of normal liver tissue [4, 5]. The ability of MVI is important to guide surgical management in patients with HCC for selection of the type of hepatectomy AR(anatomical resection) or NAR (non-anatomical resection), width of surgical margins, and use of adjuvant or neoadjuvant therapy. Surgical resection with a wide margin could better eradicate MVI adjacent to the tumor, the decision to resect with a wide margin has to be made before treatment. Although postoperative supplementation with remedy TACE treatment can improve the postoperative survival time and recurrence time, it still lags behind in the progress of the tumor, with poor long-term efficacy [6, 7].

MVI is a histopathological diagnosis, although imaging techniques, such as ultrasonography, computed tomography (CT), and magnetic resonance imaging (MRI), are widely used to assess preoperative examinations of patients with HCC. However, it is difficult to effectively detect MVI, which requires to be diagnosed by postoperative histopathology. MVI more directly reflects the ability of tumor invasion and metastasis compared with morphological indicators [8]. It can represent the degree of malignancy of the tumor, and is an independent predictor of both disease-free survival (DFS) and overall survival (OS) [9]. It is vital in making reasonable surgical planning and selecting suitable treatment options for postoperative patients [10]. A study by Jang reported that the recurrence rate for patients with MVI was higher compared with the rate in those without MVI, and the recurrence time was earlier [11]. Therefore, an accurate preoperative prediction of the MVI status is critical to the choice of HCC therapy for impeding recurrence and improving the outcome.

Chronic inflammatory response has already been considered as the main cause of HCC, such as of HBV and HCV. In this study, chronic hepatitis (especially HBV infection) was the most common cause of HCC. A decade ago, some clinical medical institutions were already aware of the predictive value of systemic inflammatory response in the outcome of patients undergoing resection for HCC [12]. Both neutrophils and lymphocytes were the most important part of white blood cells in systemic inflammatory response and recognized as key participators in metastasis based on increasing evidence [13–15]. Neutrophil-to-lymphocyte ratio (NLR), platelet-to-lymphocyte ratio (PLR), and aspartate aminotransferase/platelet count ratio index (APRI) were convenient markers for predicting poor prognosis for HCC [16, 17]. Whether NLR, PLR, or APRI can predict the MVI of HCC is still unknown. Therefore, a retrospective analysis of the predictive value of preoperative NLR and possible clinical parameters to MVI in patients with HCC was performed in this study.

## RESULTS

### Patient characteristics

This study included 157 patients (114 males and 43 females) who had undergone a curative resection for HCC. The tumor diameter was less than or equal to 5 cm. The average age was 54.9 ± 9.3 years (ranging from 35–77 years). A total of 62.4% (98/157) patients developed recurrence, and 42.0% (66/157) patients died during the follow-up. Hepatitis B surface antigen positivity accounted for 67.5% (106/157), and hepatitis C antibody positivity accounted for 17.2% (27/157). Moreover, 15.3% (24/157) had no evidence of hepatitis. All diagnoses were ultimately confirmed both clinically and pathologically, and MVI was also confirmed pathologically. The general clinical factors are summarized in Tables 1 and 3, and the quantitative clinical features are shown in Table 2. Among the 157 patients, 26.1% (41/157) developed MVI after operation.

**Table 1:** Univariate and multivariate analysis of clinicopathologic variables in relation to DFS and OS after curative operation

Variables		*N*	Univariate analysis		Multivariate analysis			
			DFS	OS	DFS		OS	
			*P*	*P*	HR (95% CI)	*P*	HR (95% CI)	*P*
Gender	Male	114	0.768	0.532	1.043 (0.624–1.743)	0.873	1.322 (0.706–2.473)	0.383
	Female	43						
Age(years)	< 50	49	0.314	0.626	1.054 (0.604–1.838)	0.853	1.158 (0.568–2.354)	0.690
	≥ 50	108						
ABO	A	40	0.605	0.673	1.049 (0.832–1.322)	0.687	0.887 (0.659–1.193)	0.428
	AB	17						
	B	61						
	O	39						
CEA(ng/mL)	< 5	133	0.084	0.277	0.984 (0.871–1.112)	0.790	1.082 (0.920–1.272)	0.339
	≥ 5	24						
AFP(U/mL)	< 200	110	0.000	0.848	1.000 (0.939–1.156)	0.727	1.000 (0.901–1.201)	0.495
	≥ 200	47						
CA199(U/mL)	< 39	126	0.060	0.260	1.000 (1.000–1.001)	0.111	0.233 (0.998–1.000)	0.999
	≥ 39	31						
PLR	< 115	96	0.505	0.042	0.997 (0.994–1.001)	0.197	0.994 (0.988–1.000)	0.037
	≥ 115	61						
NLR	< 2	46	0.037	0.012	0.946 (0.854–1.049)	0.293	0.943 (0.801–1.110)	0.479
	≥ 2	111						
APRI	< 1.6	144	0.755	0.418	0.539 (0.285–1.021)	0.058	0.375 (0.166–0.846)	0.018
	≥ 1.6	13						
Tumor number	1	111	0.000	0.000	1.553 (0.731–3.301)	0.253	7.675 (3.196–18.431)	0.000
	≥ 2	46						
Differentiation	well	47	0.478	0.000	1.312 (0.946–1.820)	0.104	3.413 (2.170–5.368)	0.000
	moderate	69						
	poor	41						
Size(cm)	< 3	49	0.405	0.010	0.891 (0.683–1.161)	0.392	1.002 (0.670–1.497)	0.993
	≥ 3, < 5	108						
Child-Pugh	A	130	0.767	0.911	1.179 (.544–2.553)	0.676	2.934 (1.008–8.541)	0.048
	B	27						
HBV-sAg	Positive	106	0.582	0.026	1.341 (.679–2.646)	0.398	0.878 (0.319–2.412)	0.800
	Negative	51						
HCV-Ab	Positive	27	0.789	0.196	1.418 (.626–3.212)	0.403	0.788 (0.213–2.921)	0.722
	Negative	130						
ALT	< 80	142	0.303	0.529	1.003 (0.996–1.010)	0.413	1.005 (0.997–1.014)	0.221
	≥ 80	27						
MVI	Positive	41	0.000	0.000	2.401 (1.142–5.049)	0.021	0.663 (0.292–1.507)	0.327
	Negative	116						

NLR, neutrophil / lymphocyte rates; PLR, platelet / lymphocyte rates; APRI, aspartate aminotransferase / platelet rates.

**Table 2:** Comparison of quantitative clinical factors between MVI- Negative group and MVI- Positive group

Factors	MVI- Negative		MVI- Positive		*P* value
	Mean	Standard deviation	Mean	Standard deviation	
Age (years)	55.110	9.509	54.682	8.728	0.804
Abl (g/L)	37.553	6.088	35.895	5.734	0.130
CEA (ng/mL)	3.285	2.261	2.563	1.306	0.056
AFP (μg/L)	138.155	332.423	6627.320	15838.761	0.000
CA19-9(U/mL)	93.945	520.080	185.265	795.579	0.406
Platelet (×103/mL)	167.724	71.839	175.188	66.422	0.561
Lymphocyte (×103/mL)	3.104	11.697	1.715	0.702	0.450
Neutrophil (×103/mL)	4.593	2.505	4.196	2.520	0.385
PLR	130.116	95.997	124.224	82.017	0.727
NLR	3.792	3.725	2.878	2.074	0.046
Tumor size (cm)	3.883	0.931	4.219	0.783	0.004
ALT (U/L)	45.828	50.142	69.520	94.460	0.045
APRI	0.508	0.571	0.826	1.194	0.026

NLR, neutrophil/lymphocyte rates; PLR, platelet/lymphocyte rates; APRI, aspartate aminotransferase / platelet rates.

**Table 3:** Univariate analysis of clinical characteristics according to microvascular invasion

Characteristics		MVI-Negative		MVI-Positive		Univariate analysis	
		*N* (116)	%	*N* (41)	%	*X*^2^	*P* value
Gender	Male	83	71.6	31	75.6	0.251	0.687
	Female	33	28.4	10	24.4		
Age (years)	< 50	38	32.8	11	26.8	0.496	0.559
	≥ 50	78	67.2	30	73.2		
Child-Pugh	A	98	84.5	32	78.0	0.881	0.345
	B	18	15.5	9	22.0		
CEA (ng/mL)	< 5	96	82.8	37	90.2	1.311	0.319
	≥ 5	20	17.2	4	9.8		
AFP(μg/L)	< 200	96	82.8	14	34.1	34.131	0.000
	≥ 200	20	17.2	27	65.9		
CA199 (U/mL)	< 39	93	80.2	33	80.5	0.002	1.000
	≥ 39	23	19.8	8	19.5		
PLR	< 115	68	58.6	28	68.3	1.193	0.352
	≥ 115	48	41.4	13	31.7		
NLR	< 2.0	28	24.1	18	43.9	5.713	0.017
	≥ 2.0	88	75.9	23	56.1		
ABO	A	31	26.7	9	22.0	0.468	0.926
	AB	12	10.3	5	12.2		
	B	44	37.9	17	41.5		
	O	29	25.0	10	24.4		
APRI	< 1.6	110	94.8	34	82.9	6.649	0.041
	≥ 1.6	6	5.2	7	17.1		
Differentiation	well	40	34.5	7	17.1	10.097	0.006
	moderate	53	45.7	16	39.0		
	poor	23	19.8	18	43.9		
Tumor size (cm)	< 3	42	36.2	7	17.1	5.166	0.030
	≥3, < 5	74	63.8	34	82.9		
Tumor number	single	103	88.8	8	19.5	70.192	0.000
	multiple	13	11.2	33	80.5		
ALT (U/L)	< 80	101	87.1	31	75.6	2.971	0.134
	≥ 80	15	12.9	10	24.4		

### Comparison of the clinicopathological characteristics in relationship to DFS and OS after operation

**Figure 1:**
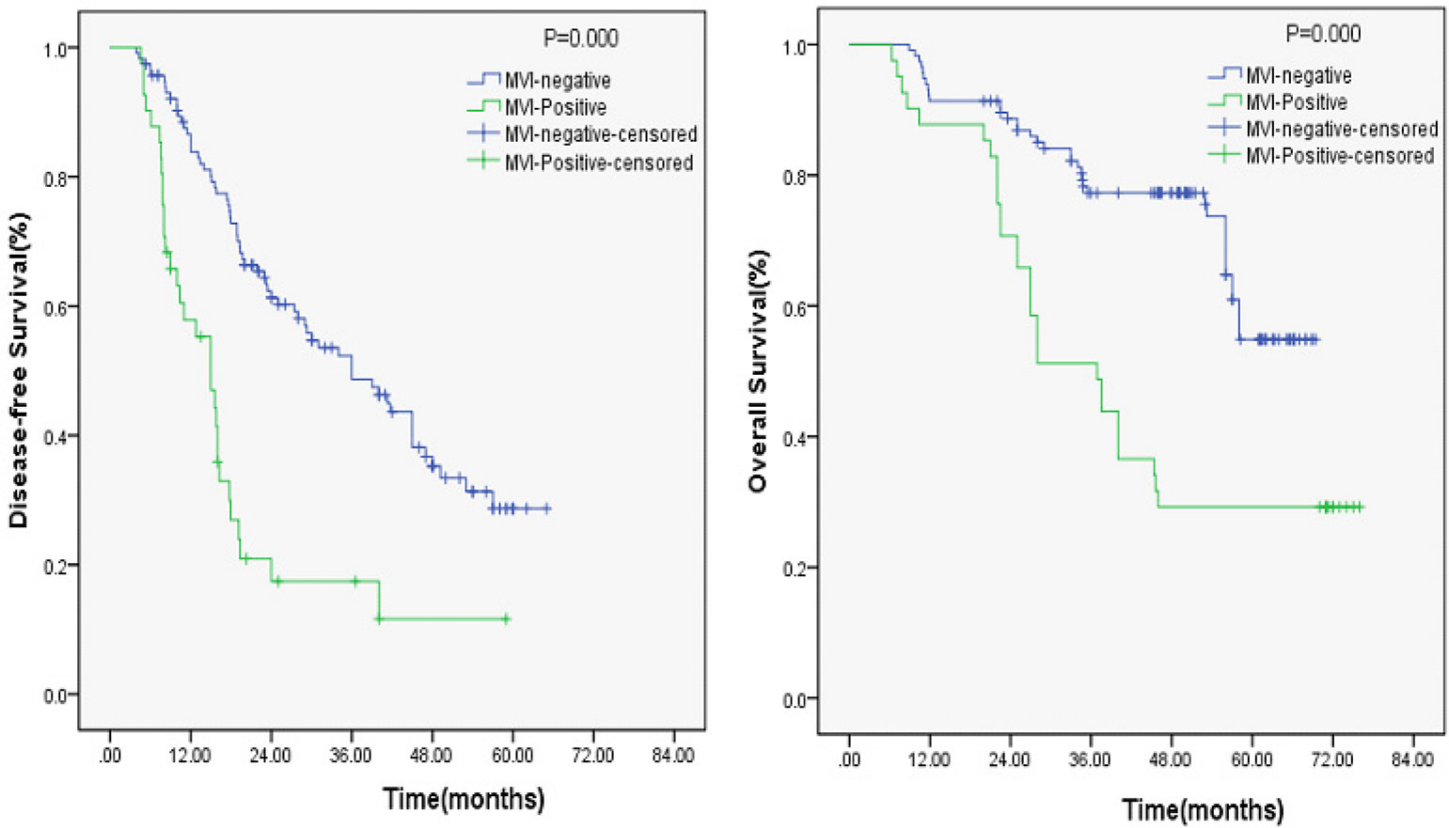
Kaplan-Meier curve for DFS and OS of patients with HCC by MVI-positive *vs* MVI-negative; MVI-positive is associated with Early recurrence (*P* = 0.000)

In the univariate analysis, NLR (*P* = 0.017), MVI (*P* = 0.000), and tumor number (*P* = 0.000) were significant prognostic factors for DFS (Table 1), whereas APRI, PLR, and AFP were not significant predictors of DFS (*P* > 0.05; Table 1). In the multivariate analysis, MVI (*P* = 0.021) was also a significant predictor of DFS (Table 1). However, MVI was not a poor predictor of OS as identified using multivariate analysis. Both univariate and multivariate analyses showed that PLR, tumor number, and tumor differentiation were significantly associated with OS in patients with HCC who underwent curative resection. The association between MVI and DFS, and MVI and OS after surgery estimated using the Kaplan–Meier curve (Figure 1) showed that MVI was an independent predictor of DFS in patients with HCC who underwent curative resection.

### Determination of the cut-off values

**Figure 2:**
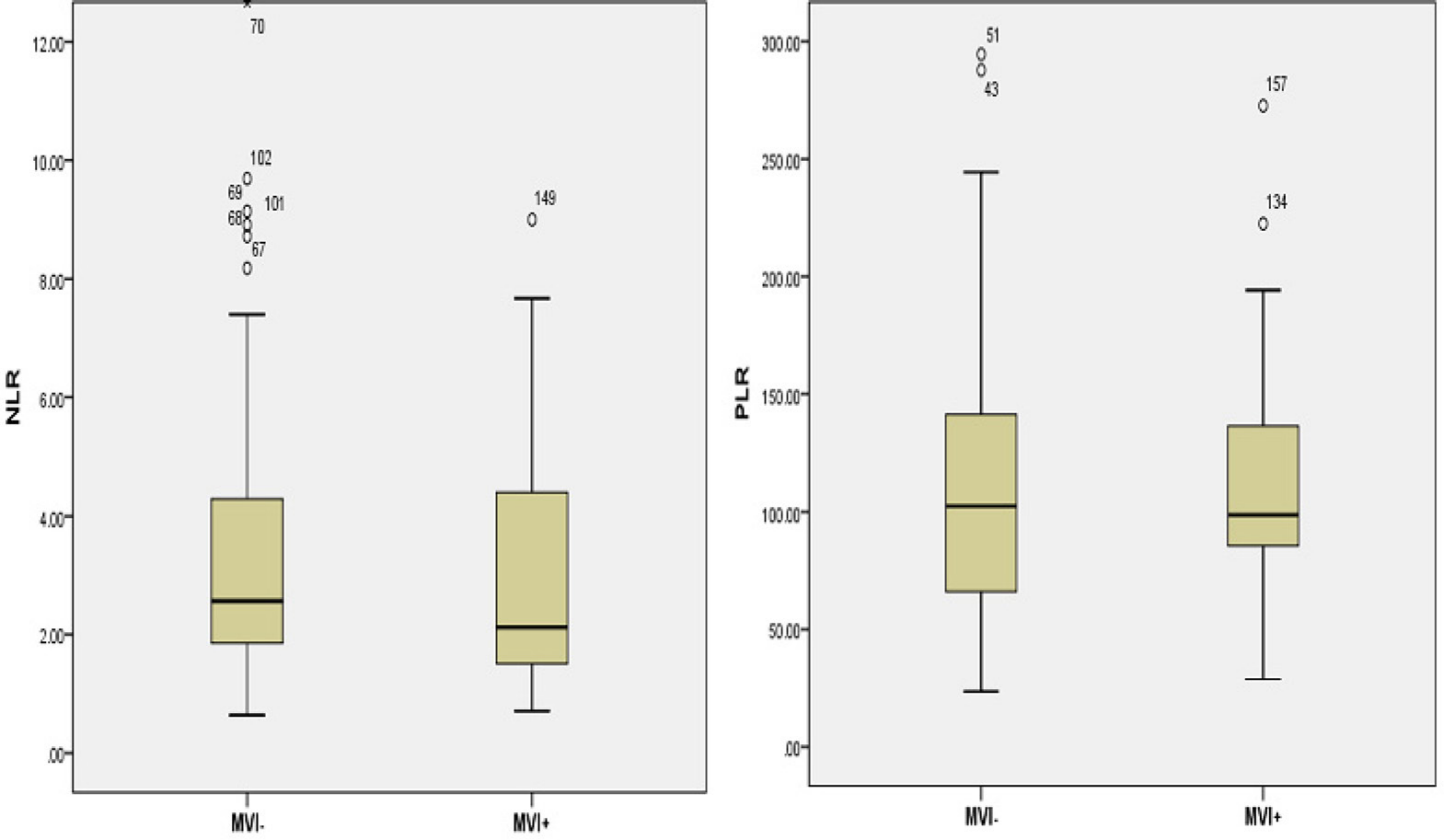
Distributions of NLR A. and PLR B. between MVI+ and MVI−

According to the receiver operating characteristic (ROC) curve plotted (Figure 2), the cut-off value of PLR, NLR, and APRI for MVI was set to 115, 2.0, and 1.6, respectively. Thus, the patients were dichotomized into groups of “high PLR (≥ 115)” and “low PLR (< 115),” or ”high NLR (≥ 2)” and “low NLR (≥ 2),” or “high APRI (≥ 1.6)” and “low APRI (≥ 1.6).”

### Preoperative NLR, PLR, APRI, and clinical parameters of HCCs with and without MVI

**Figure 3:**
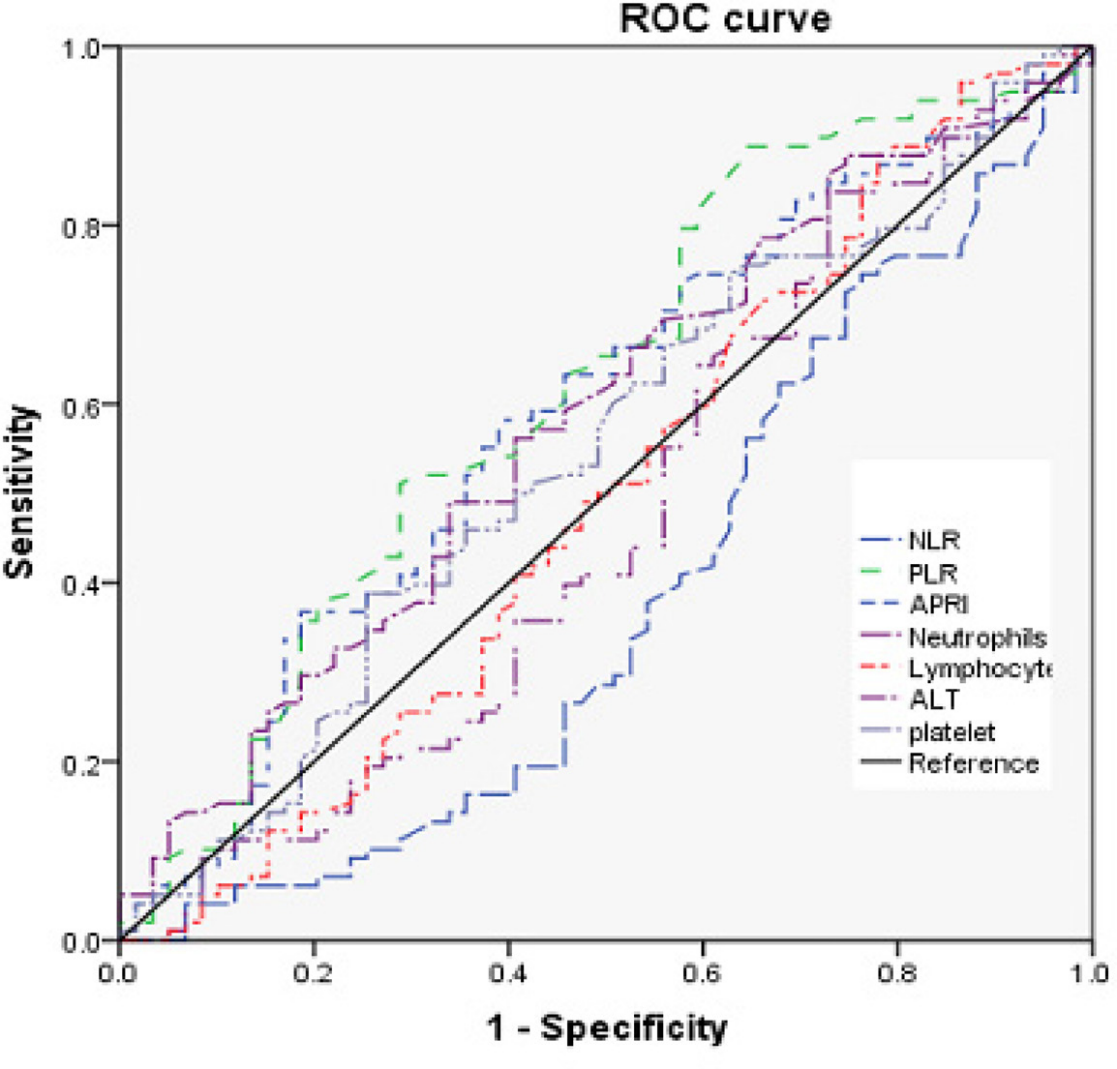
ROC curves for systemic inflammatory response markers in patients with HCC according to MVI-positive (NLR, neutrophil-tolymphocyte ratio; PLR, platelet-to-lymphocyte ratio; ROC, receiver operating characteristic)

The neutrophil and lymphocyte counts were not significantly different between patients with HCC having MVI and those not having MVI (Table 2). However, NLR (*P* = 0.045), AFP (*P* = 0.000), tumor size (*P* = 0.004), *alanine* aminotransferase (ALT; *P* = 0.045), and APRI (*P* = 0.026) were significantly higher in patients with HCC having MVI (Table 2 and Figure 3). The χ^2^ test was further used to evaluate these variables. Table 3 shows that NLR (χ2 = 5.713, *P* = 0.007), AFP (χ^2^ = 34.131, *P* = 0.000), APRI (χ^2^ = 6.649, *P* = 0.041), and tumor number (χ^2^ = 70.192, *P* = 0.000) were statistically significant, signifying that the ability of preoperative NLR values to differentiate MVI was more powerful than that of individual indicators of lymphocytes, neutrophils, and platelets.The serum levels of NLR, APRI, and AFP were statistically higher in the MVI-positive group (both *P* < 0.05) (Table 3). The tumor size was statistically bigger in the MVI-positive group (*P* = 0.03). No significant differences were observed between the two groups about other quantitative clinical characteristics, including age, carbohydrate antigen 199 (CA199), and carcinoembryonic antigen (CEA).

### Univariate and multivariate analyses of MVI-related factors

Univariate analysis indicated that NLR (χ^2^ = 5.713, *P* = 0.017), AFP (χ^2^ = 34.131, *P* = 0.000), APRI (χ^2^ = 6.649, *P* = 0.041), differentiation (χ^2^ = 10.097, *P* = 0.006), tumor size (χ^2^ = 5.166, *P* < 0.030), and tumor number (χ^2^ = 70.192, *P* = 0.000) showed significant differences according to the MVI existence (Table 3). No significant association was found between the MVI-negative and MVI-positive groups with respect to gender, age, CEA, CA199, ABO blood type, Child–Pugh score, PLR, and ALT. The multivariate analysis results showed that (Table 4) four of these differed significantly (*P* < 0.05). Finally, NLR [hazard ratio (HR), 1.705; 95% confidence interval (CI), 0.467–6.232; *P* = 0.022], AFP (HR, 1.012; 95% CI, 1.003–1.021; *P* =.007), tumor size (HR, 1.025; 95% CI, 0.989–1.062; *P* = 0.028), and tumor number (HR, 2.738; 95% CI, 1.151–6.515; *P* = 0.008) were identified to be independent predictive indicators of MVI.

**Table 4:** Results of the clinicopathological for HCC with MVI-Positive by multivariate logistic analyses

Parameters		Hazard ratio	95% CI	*P* value
AFP (μg/L)	< 200	2.012	2.003–2.021	0.007
	≥ 200			
Tumor number	single	2.738	1.151–6.515	0.008
	multiple			
Differentiation	well			0.089
	moderate	0.028	0.000–13.102	0.255
	poor	4.043	0.029–562.704	0.579
Tumor size (cm)	< 3	0.011	0.000–0.615	0.028
	≥ 3, < 5			
PLR	< 115	1.048	1.006–1.092	0.025
	≥ 115			
NLR	< 2.0	1.295	1.104–1.842	0.022
	≥ 2.0			
APRI	< 1.6	0.179	0.001–29.170	0.508
	≥ 1.6			

## DISCUSSION

The host inflammatory response is correlated with the occurrence and development of HCC. As a poor prognostic indicator, MVI is an independent risk factor for the survival and recurrence of patients with HCC [9, 11]. The results of the present study showed that 26.1% (41/157) patients with HCC had MVI when diagnosed, and DFS of the MVI-negative group was significantly higher than that of the MVI-positive group. The important mechanism underlying this was the early spread of tumor cells through blood vessels [18]. The MVI preoperative prediction is still an unresolved problem. About 15%–35% cases in small HCC were with MVI [19]. MVI was an independent predictor of DFS in patients with HCC who underwent a curative resection. Moreover, it was a key preoperative factor for selecting comprehensive treatment for patients with HCC [20]. Therefore, preoperative predictive indicators of MVI in patients with HCC are essential.

The present study demonstrated that NLR and MVI were closely related (*P* = 0.045), and 84.7% (113/157) patients were infected with HBV and HCV, consistent with many previous studies. Chronic inflammation was correlated with the occurrence and development of HCC [21, 22]. Neutrophils maintain the tumor microenvironment, exert protumoral functions, and enhance tumor cell invasion and metastasis, angiogenesis, and extracellular matrix remodeling in cancers [13], such as colorectal cancer, pancreatic carcinoma, and lung cancer, an thus are important in tumorigenesis and progression [23, 24]. Studies have shown that neutrophils are crucial in tumor development and metastatic progression [14, 25]. Catharina Hagerlinga and Zena Werba clarified that neutrophils create a mutagenic environment capable of initiating and promoting tumor development [26]. Therefore, it could be concluded that neutrophils might mediate MVI.

AFP is a common serum marker for tumor. It is the most useful single biomarker for diagnosing HCC [27, 28] and gynecological tumors [29]. The usefulness of AFP as a biomarker for detecting HCC was examined based on previous studies, demonstrating that elevated levels of AFP in patients with HCC was a risk factor for the development of HCC [30]. Data in the present study indicated that AFP (*P* = 0.001) was an independent predictive indicator of MVI, consistent with many previous studies [31]. Therefore, AFP might be a promising, noninvasive, MVI-associated biomarker for HCC.

This study had some limitations. First, it was a retrospective, hospital-based, single-institution, and not a population-based, study. However, this limitation was outweighed by its strength. No ascertaining bias was introduced for misdiagnosis because all patients had both pathologically and clinically confirmed MVI and HCC. Second, the loss of information on some quantifiable factors and the small size of patients did not allow the estimation of the magnitude of HCC risk for MVI associated with these factors. Although this study had a relatively small sample size, the incidence of MVI was high in HCC after operation, and given that this was the major focus of this study, the sample size was probably adequate.

In conclusion, this study showed that MVI was an independent predictor of DFS in patients with HCC who underwent a curative resection. NLR and AFP could be used as a convenient, reliable, and economical predictive means to distinguish between patients with and without MVI in HCC, which can be useful for further planning of comprehensive treatment.

## MATERIALS AND METHODS

### Ethical statement

This study was approved by the Clinical Ethics Committee of Chifeng, Inner Mongolia Medical University. The patients’ data were analyzed anonymously because written consent was not obtained from all participants.

### Study population and design

A total of 157 patients who had undergone a primary attempt of curative resection for HCC were included in this study. All participants were enrolled from Chifeng Municipal Hospital, Inner Mongolia Medical University institute of clinical from February 2010 to December 2014. There is Exclusion criteria included: presence of extrahepatic malignancy and previously treated for any type of malignancy before HCC was diagnosed;existing second malignancy;all other conditions with elevated AFP rather than liver disease. All diagnoses were confirmed by pathological examination. All surgical specimens were evaluated pathologically to determine the extent of tumor differentiation, MVI, and surgical margins following surgery.

Microscopic vascular invasion was diagnosed by the presence of clusters of cancer cells floating in the vascular space fine by endothelial cells on histopathologic examination of the resected specimen. Surgical specimens were collected and evaluated by hematoxylin and eosin (HE). A biochemical examination of blood, including a complete blood count, was generally performed 3–7 days before surgery. According to the previous studies, the preoperative NLR, PLR, and APRI were calculated as follows: preoperative NLR = the neutrophil count/lymphocyte count, preoperative PLR = platelet count/lymphocyte count, and preoperative APRI = aspartate aminotransferase count/platelet count.

Radical resection was defined as complete tumor clearance both macroscopically and histologically. All patients were followed up by telephone or as outpatient. Within 2 years after surgery, regular monitoring of serum (AFP, CEA, CA199), liver function every 3 months, liver ultrasound, and chest x-ray investigations were performed, besides contrast CT and/or MRI every 6 months, when suspected for tumor recurrence or metastasis. Tumor recurrence was defined by clinical, radiological diagnosis. Intrahepatic recurrence or metastasis was determined by two imaging examinations with typical performance. The DFS was the time of operation to patients with tumor recurrence time. OS was the time of operation to the patient’s death or the last follow-up time. Remedial treatments were selected, including reoperation, TACE, RFA, sorafenib, and radiotherapy, when the recurrence was determined.

### Statistical analysis

Statistical analysis was performed using the SPSS 20.0 statistical software (SPSS, IL, USA). A *P* value < 0.05 was considered statistically significant. The Mann–Whitney U-test and a box plot were used to describe the normality of each continuous parameter’s distribution. Quantitative values were reported in the form of means ± standard deviation. Associations between clinical and histopathological parameters with OS, DFS, and MVI were analyzed using the Kaplan–Meier curves and compared using the log-rank test. Univariate and multivariate Cox regression analyses were performed to determine the effects of possible prognostic factors on MVI after curative operation. HRs estimated from the Cox analysis were shown as relative risks with corresponding 95% CIs. The ROC curve was used to estimate the performance of NLR, PLR, and APRI. The χ^2^ test was used for the univariate analysis of MVI. Associations between clinical and histopathological parameters with MVI were evaluated by both univariate and multivariate logistic regression analyses.
